# Simultaneous Bilateral Renal Revascularization in an Elderly Patient With Recurrent Flash Pulmonary Edema: A Case Report

**DOI:** 10.7759/cureus.101079

**Published:** 2026-01-08

**Authors:** Adalberto Teixeira da Matta Flora Neto, Gabriel Valdisser Jaculi Teixeira Bento, Laura Couto de Oliveira Azevedo, Isabela Martins Rodrigues, Julliana Silva Luiz, João Paulo Ferreira Campos, Luiz Guilherme Amaral Morisson, João Lucas O'Connell, Beatriz M Silva, Thaís M Silva

**Affiliations:** 1 Department of Cardiology, Universidade Federal de Uberlândia, Uberlândia, BRA; 2 Department of Cardiology, Mário Palmério Hospital Universitário, Uberaba, BRA

**Keywords:** chronic kidney disease, pulmonary edema, renal artery angioplasty, renal artery stenosis, secondary hypertension

## Abstract

Renal artery stenosis (RAS) is a well-established cause of secondary hypertension and progressive chronic kidney disease (CKD), and severe bilateral disease may precipitate recurrent episodes of acute heart failure, including flash pulmonary edema, particularly in elderly patients with multiple cardiovascular comorbidities. This report highlights the diagnostic approach, therapeutic strategy, and outcomes in a complex patient with multiple comorbidities. A 78-year-old woman with multiple comorbidities, including hypertension, insulin-dependent diabetes mellitus, dyslipidemia, asthma, peripheral arterial disease, spherocytosis, and chronic kidney disease, presented with recurrent episodes of acute pulmonary edema (APE) initially attributed to heart failure with preserved ejection fraction (HFpEF). Transthoracic echocardiography showed grade II diastolic dysfunction, pulmonary hypertension, and mild valvular disease. Coronary catheterization excluded significant obstructive coronary artery disease, whereas renal angiography revealed severe bilateral renal artery stenosis (right: 65%, left: 80%-90%), associated with worsening renal function and refractory hypertension. Given the high-risk clinical phenotype, the patient underwent bilateral renal artery angioplasty with stent placement, following nephroprotective measures due to the elevated risk of contrast-induced nephropathy. The procedure was uneventful with good angiographic results. At one-year follow-up, no further episodes of pulmonary edema occurred, renal function improved, blood pressure was well controlled, and renal arteries remained patent on ultrasound. This case highlights the importance of evaluating renal artery stenosis in patients with sudden or recurrent acute pulmonary edema, refractory hypertension, or progressive renal dysfunction, particularly in the presence of multiple comorbidities. Bilateral renal revascularization was effective in stabilizing renal function and improving blood pressure control; however, long-term follow-up remains essential.

## Introduction

Renal artery stenosis (RAS) is a recognized cause of secondary hypertension and progression of chronic kidney disease (CKD), particularly in elderly patients with systemic atherosclerosis and multiple cardiovascular comorbidities [1]. The atherosclerotic form is the most prevalent and reflects a diffuse vascular process commonly associated with diabetes mellitus and dyslipidemia. In this setting, alterations in lipoprotein metabolism contribute to atherogenesis and are linked to increased cardiovascular risk [2]. While unilateral RAS is relatively common, hemodynamically significant bilateral disease or involvement of a solitary functioning kidney is less frequent and is often associated with greater clinical instability, worsening renal function, and challenges in blood pressure control [1,3]. This case demonstrates the successful use of simultaneous bilateral renal revascularization in an elderly high-risk patient, highlighting its potential applicability in selected cases.

Impaired renal perfusion in significant RAS leads to the activation of the renin-angiotensin-aldosterone system, resulting in sodium and water retention, vasoconstriction, and increased cardiac workload [3]. These mechanisms may be particularly relevant in patients with long-standing hypertension and diastolic dysfunction, contributing to the development of heart failure with preserved ejection fraction (HFpEF) [4]. In this context, recurrent episodes of acute pulmonary edema (APE), classically described as Pickering syndrome, represent an important clinical manifestation of severe bilateral RAS and should raise suspicion for a renovascular etiology in susceptible patients [5].

The role of renal artery revascularization in atherosclerotic RAS remains debated. Randomized trials comparing stenting with optimized medical therapy did not demonstrate a clear benefit in broadly selected populations [6,7]. Randomized controlled trials comparing renal artery stenting with optimized medical therapy failed to demonstrate a clear benefit in broadly selected populations [6,7]. However, these trials largely excluded patients with high-risk clinical features. In contrast, observational studies, contemporary case series, and recent scientific statements (2025-2026) have suggested potential clinical benefit of revascularization in carefully selected high-risk patients, particularly those with bilateral disease, recurrent flash pulmonary edema, rapidly progressive renal dysfunction, or refractory hypertension. Current scientific statements and hypertension guidelines therefore recommend a selective and individualized approach, suggesting that revascularization may be considered in patients with clear clinical consequences of renal ischemia [8,9]. Within this framework, the present case illustrates how targeted investigation of secondary and potentially reversible causes of hypertension and heart failure, specifically severe bilateral RAS, can inform management decisions and be associated with favorable clinical outcomes, even in elderly patients with preserved systolic function and multiple comorbidities [9].

## Case presentation

A 78-year-old woman with relevant comorbidities, hypertension, asthma, peripheral arterial disease, dyslipidemia, insulin-dependent diabetes, chronic renal failure, and spherocytosis, presented to the emergency department. Fifteen days prior, she began experiencing symptoms initially interpreted as heart failure with preserved ejection fraction (HFpEF), with functional class III dyspnea, episodes of acute pulmonary edema, poorly controlled hypertension, and anginal chest pain; on physical examination, the only abnormality was a 2+/6+ pancardiac systolic murmur. Transthoracic echocardiography at the onset of symptoms showed a left ventricular ejection fraction (LVEF) of 69%, slight left atrial enlargement (indexed volume: 35), grade II left ventricular diastolic dysfunction, mild to moderate mitral regurgitation, moderate tricuspid regurgitation, and pulmonary hypertension (pulmonary artery systolic pressure: 48 mmHg).

**Figure 1:**
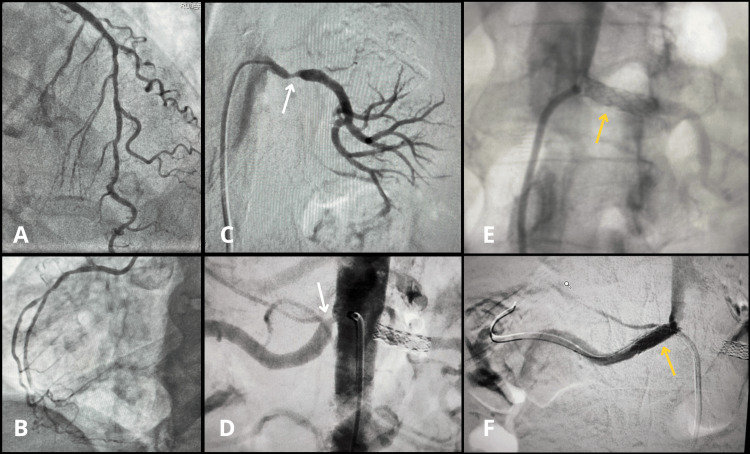
Angiographic findings of the coronary and renal arteries (A) Left coronary angiography (cranial posteroanterior view) showing mild stenoses and tortuosity in the left anterior descending artery and diagonal branches. (B) Right coronary angiography showing a non-dominant vessel with mild stenoses. (C) Left renal arteriography demonstrating significant proximal stenosis (white arrow). (D) Digital subtraction abdominal aortography showing significant ostial stenosis of the right renal artery (white arrow) alongside the already stented left renal artery. (E) Left renal angioplasty showing stent placement covering the ostium and proximal segment (yellow arrow). (F) Right renal angioplasty showing stent placement covering the ostium and proximal segment (yellow arrow).

Two weeks after the onset, due to suspected angina, catheterization with coronary angiography (and superselective secondary branch angiography) was performed, revealing diffuse atherosclerosis and long/moderate stenosis of 50% in proximal segments of the left anterior descending artery (LAD), in addition to diffuse atherosclerosis and slight stenosis in the mid-segment of the right coronary artery (RCA). In the associated renal evaluation, right renal arteriography showed moderate proximal stenosis (65%) with tortuosity, and left renal arteriography showed significant proximal stenosis (80%-90%) in the main renal artery, concluding bilateral renal artery stenosis and non-oliguric Kidney Disease: Improving Global Outcomes (KDIGO) stage 2 acute kidney injury (AKI) (Figure 1). Two days after the angiography, conservative renal management was chosen, with no indication/benefit for renal replacement therapy; antihypertensive treatment was maintained, as the refractory hypertension had not yet been fully addressed.

Based on the Mehran score (15 points), the risk of worsening renal function was estimated at 26% and the risk of dialysis at 1.09%, with angiotomography not contraindicated for stenosis assessment. Nephroprotection was performed with a bicarbonate solution (5% glucose solution 850 mL + 8.4% sodium bicarbonate IV 150 mL), with a lower volume (3 mL/kg one hour before and 1 mL/kg/hour for six hours after), as well as daily monitoring of renal function and monitoring of rigorous blood pressure. Nephrotoxic agents (especially anti-inflammatory drugs) and angiotensin-converting enzyme (ACE) inhibitors/angiotensin-receptor blockers (ARBs) were avoided, and antibiotics were adjusted according to creatinine clearance and whether or not hemodialysis was performed. One week after angiography, the patient presented with creatinine of 1.18 mg/dL, glomerular filtration rate of 46 mL/min/1.73 m², and urea of 33 mg/dL. Consequently, the decision was made to perform right deep femoral artery arterioplasty with a vascular closure device, with bilateral renal arteriography using digital subtraction and invasive blood pressure monitoring, with dilation of the stenosed segments, without complications, and the patient was referred to the coronary care unit (CCU) for observation/monitoring and optimization of coronary artery disease treatment. The bilateral approach in the same procedure was chosen due to difficulty in blood pressure control, progressive worsening of renal function, and recent episodes of sudden acute pulmonary edema (APE). Eight days after bilateral renal artery revascularization, laboratory tests showed no acute changes in renal function, with creatinine of 1.21 mg/dL, glomerular filtration rate of 45 mL/min/1.73 m², and urea of 41.8 mg/dL (Table 1).

**Table 1:** Renal function parameters at different clinical time points eGFR: estimated glomerular filtration rate

Parameter	Reference range	Pre-angiography	7 days post-angiography	Post-revascularization (8 days)
Creatinine (mg/dL)	0.50-1.00 (adult female)	2.88	1.18	1.21
eGFR (mL/min/1.73 m²)	>60	15	46	45
Urea (mg/dL)	16.6-48.5	144	33	41.8

Approximately two months later, an ultrasound of the urinary tract showed a right kidney measuring 10.7×5.7×4.4 cm (141.3 cm³) and a left kidney measuring 10.6×4.5×4.4 cm (110.0 cm³), without hydronephrosis or lithiasis, with preserved cortical thickness and corticomedullary differentiation bilaterally, suggesting possible incipient chronic parenchymal nephropathy.

**Figure 2:**
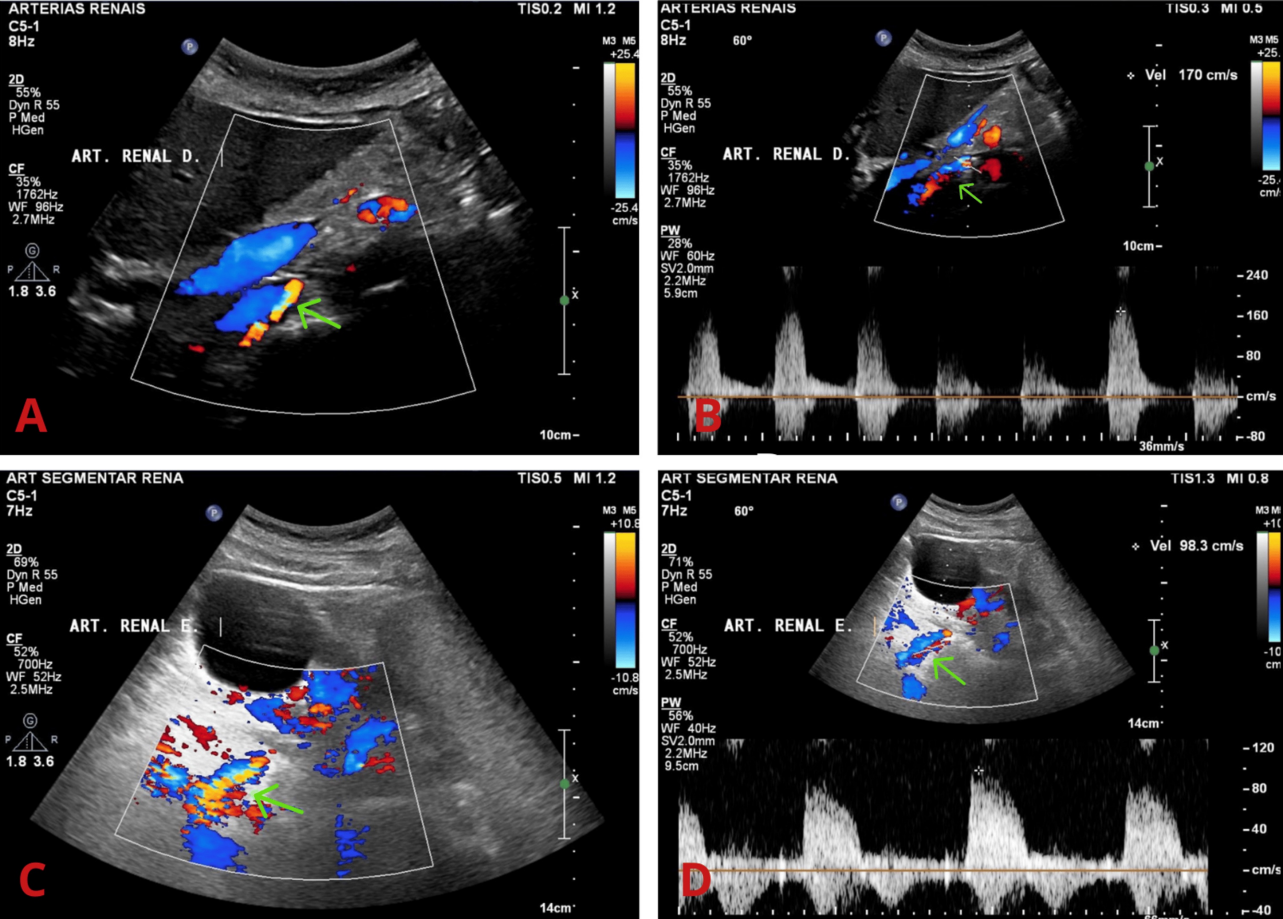
Renal ultrasound demonstrating adequate blood flow in the renal arteries (A) Color Doppler ultrasound demonstrates flow in the topography of the right renal artery (green arrow), with no evidence of hemodynamically significant stenosis along its course. The stent image was not adequately visualized on 2D imaging. (B) Peak systolic velocity measured at 170 cm/s in the proximal renal artery segment (reference value: <200 cm/s). (C) Color Doppler ultrasound demonstrates flow in the topography of the left renal artery (green arrow), with no evidence of hemodynamically significant stenosis along its course. The stent image was not adequately visualized on 2D imaging. (D) Peak systolic flow velocity measured at 98 cm/s in the proximal segment of the left renal artery (reference value: <200 cm/s).

During the one-year follow-up, the patient presented no further episodes of sudden pulmonary edema; there was improvement in renal function and adequate blood pressure control, with a reduction in the number and dose of routinely used antihypertensive medications. Postoperative renal ultrasound showed good flow through the renal arteries, which were patent (Figure 2).

## Discussion

Atherosclerotic renal artery stenosis is a manifestation of systemic atherosclerosis and commonly affects older patients with multiple cardiovascular comorbidities. While mild or unilateral RAS is often clinically silent, severe bilateral disease can be associated with a distinct high-risk presentation characterized by abrupt heart failure decompensations, accelerated decline in kidney function, and difficult-to-control hypertension [1].

Recurrent and rapidly developing pulmonary edema in a patient with hypertension and atherosclerotic renovascular disease (ARVD) should raise suspicion for Pickering syndrome. This syndrome occurs when severe renal hypoperfusion triggers sustained activation of the renin-angiotensin-aldosterone system (RAAS) [5]. This is most commonly caused by bilateral atherosclerotic RAS or stenosis of a solitary functioning kidney, leading to abrupt sodium and water retention and increased systemic vasoconstriction. These rapid increases in preload and afterload are poorly tolerated in patients with hypertensive remodeling and diastolic dysfunction (an HFpEF substrate). In this setting, small volume shifts can disproportionately raise left-sided filling pressures and trigger flash pulmonary edema despite preserved LVEF [5]. This volume-sensitive physiology is accentuated when contralateral natriuresis is limited, which helps explain the classic association with bilateral disease and why prompt recognition of a renovascular driver can be clinically decisive.

There is a strong mechanistic rationale for a renovascular driver in flash pulmonary edema. Even so, renovascular contributors remain frequently under-recognized in clinical practice. One reason is the broad extrapolation of neutral findings from landmark renal revascularization trials in heterogeneous atherosclerotic RAS populations [8]. These studies often included patients without clearly flow-limiting disease and generally did not represent the unstable phenotype of recurrent flash pulmonary edema. In practice, this can lead to recurrent pulmonary edema being managed as HFpEF exacerbation alone. In addition, RAAS blockade, often beneficial in cardiovascular disease, can complicate management in hemodynamically important bilateral RAS [10].

Accordingly, clinicians should actively evaluate a renovascular driver in patients with pulmonary edema who present with (i) sudden or recurrent flash pulmonary edema disproportionate to the degree of systolic dysfunction, (ii) severe, resistant, or abruptly worsening hypertension after a previously stable course, and/or (iii) worsening renal function, particularly when deterioration follows therapies that modulate the renin-angiotensin system [8]. Additional supportive clues include a background of systemic atherosclerosis and suggestive imaging findings such as renal size asymmetry or parenchymal changes on ultrasound [11].

Our patient exhibited this pattern: she developed recurrent flash pulmonary edema and difficult-to-control hypertension over 15 days despite preserved LVEF (69%) with grade II diastolic dysfunction (pulmonary artery systolic pressure: 48 mmHg), and coronary angiography showed no culprit severe lesion. Renal angiography demonstrated bilateral significant RAS (right: 65%, left: 80%-90%) with non-oliguric AKI, KDIGO stage 2, supporting a renovascular-driven management strategy.

Management of atherosclerotic RAS includes aggressive medical optimization, blood pressure and volume control, lipid-lowering, antiplatelet therapy when indicated, and intensive cardiovascular risk modification, regardless of whether revascularization is pursued [9,10]. Contemporary scientific statements and clinical practice documents support considering endovascular intervention in selected high-risk presentations, particularly recurrent flash pulmonary edema or refractory heart failure, when renovascular disease is angiographically severe or otherwise clinically consequential [8,10].

Large randomized trials showed no overall advantage of routine stenting over medical therapy for major blood pressure, renal, or cardiovascular outcomes in broadly selected atherosclerotic RAS populations [6,7]. However, these trials had important limitations and did not adequately represent unstable high-risk phenotypes such as recurrent flash pulmonary edema [8,10]. Accordingly, a presentation-driven approach may be considered in carefully selected patients. Observational data suggest that percutaneous transluminal renal artery angioplasty (PTRA) with or without stenting may be associated with cardiorenal benefit in high-risk ARVD phenotypes such as flash pulmonary edema, resistant hypertension, or rapid loss of kidney function [10].

In this case, the decision to proceed with bilateral renal endovascular revascularization was driven by the combination of recurrent pulmonary edema, difficulty controlling blood pressure, and renal function instability in the setting of significant bilateral disease. Contrast risk was addressed with stratification (Mehran score: 15) and nephroprotection, with close laboratory surveillance and avoidance of nephrotoxins and renin-angiotensin system blockade during the acute phase. The post-intervention course, no further episodes of sudden pulmonary edema over one year, improved blood pressure control with fewer antihypertensive agents, and patency on follow-up ultrasound, supports the clinical plausibility of benefit in this high-risk phenotype [8].

## Conclusions

This case highlights that the Pickering phenotype may masquerade as HFpEF-like decompensation with preserved LVEF and that identifying a renovascular driver can meaningfully change the clinical course. In patients with recurrent flash pulmonary edema and difficult-to-control hypertension, particularly when presentations are abrupt and disproportionate to systolic impairment, an active search for atherosclerotic RAS and careful phenotyping by laterality and severity can guide individualized decisions.

Although conclusions are limited by the single-patient design and potential confounding from concurrent medical optimization, causality cannot be inferred, and generalizability is limited. The sustained absence of pulmonary edema recurrence with improved blood pressure control after revascularization supports a presentation-guided approach rather than a uniform extrapolation from unselected trial populations to unstable high-risk presentations. Ongoing surveillance for restenosis and aggressive secondary prevention remain essential given the systemic nature of atherosclerosis.
